# Lifestyle Habits and Subjective Memory Complaints in Older Users of the Terrapino Mobile Application

**DOI:** 10.1093/geroni/igaf122.2808

**Published:** 2025-12-31

**Authors:** Raena Nolan, Ross Andel, Jan Pavlik, Jakub Hort

**Affiliations:** Arizona State University, Phoenix, Arizona, United States; Arizona State University, Arizona State University, Arizona, United States; AlzheimerChain Foundation, Prague, Hlavni mesto Praha, Czech Republic; Charles University, Prague, Central Bohemian, Czech Republic

## Abstract

**Background/objective:**

Subjective memory complaints (SMCs), such as difficulty recalling names or recent events despite otherwise normal cognition, can affect daily functioning and quality of life. We examined the association between lifestyle and SMCs while considering physical and mental health.

**Methods:**

Participants were 3,789 dementia-free adults aged 60+ years with complete data who used Terrapino, a free mobile application designed to promote cognitive health, launched in December 2022. SMCs were measured with 8 yes/no items on memory-related issues. Composites of physical activity (walks, exercise, sports measured), mental activity (reading, board games, mediation etc.), and health diet (nuts, fish, vegetables, berries, meat consumption [reversed]), were measured as never, sometimes, or frequently, and z scored. Average hours of sleep/day (7-8 vs. not) were also assessed.

**Results:**

Mean age was 69.6±6.4 (60-103) years, 71% were women; 85% had at least high school education, and 43% were college educated. In an ordinal logistic regression adjusted for age, sex, and education, higher physical activity (Estimate=-0.09, p=.004) and mental activity (Estimate=-0.07, p=.025), healthier diet (Estimate=-0.21, p<.001), and getting 7-8 hours of sleep/day (Estimate=-0.32, p<.001) were all associated with lower SMCs. Adding comorbidity to the model explained away the physical activity-SMCs link (p=.088); adding hearing problems had no effect, and adding ability to manage stress explained the association between physical (p=.364) and mental (p=.057) activities and SMCs, while other associations remained significant.

**Discussion:**

Lifestyle, particularly sleep, relates to memory complaints. Some associations reflected ability to manage stress.

